# Supplementary material for: Proprioceptive feedback determines visuomotor gain in Drosophila
Source: R Soc Open Sci. 2016 Jan 13;3(1):150562. doi: 10.1098/rsos.150562 (PMC4736939; doi:10.1098/rsos.150562)
Supplement: Electronic Supplemental Material [file rsos150562supp1.pdf]

Electronic Supplemental Material (ESM)

a) Raw data traces

Raw data traces show left and right wingbeat amplitude of fruit flies flying under closed-loop visual feedback inside a virtual-reality flight simulator (figure S1). The animals actively stabilize the azimuth position of a vertical black stripe in their frontal fields of view. In these experiments, the flies were not faced with a yaw velocity bias and there was no moving visual background pattern. The traces highlight the impact of unilateral laser heat treatment of the wing nerve with 35 (blue) and 90 mW (green) and the impact of bilateral haltere immobilization (red), compared to control flies (black).

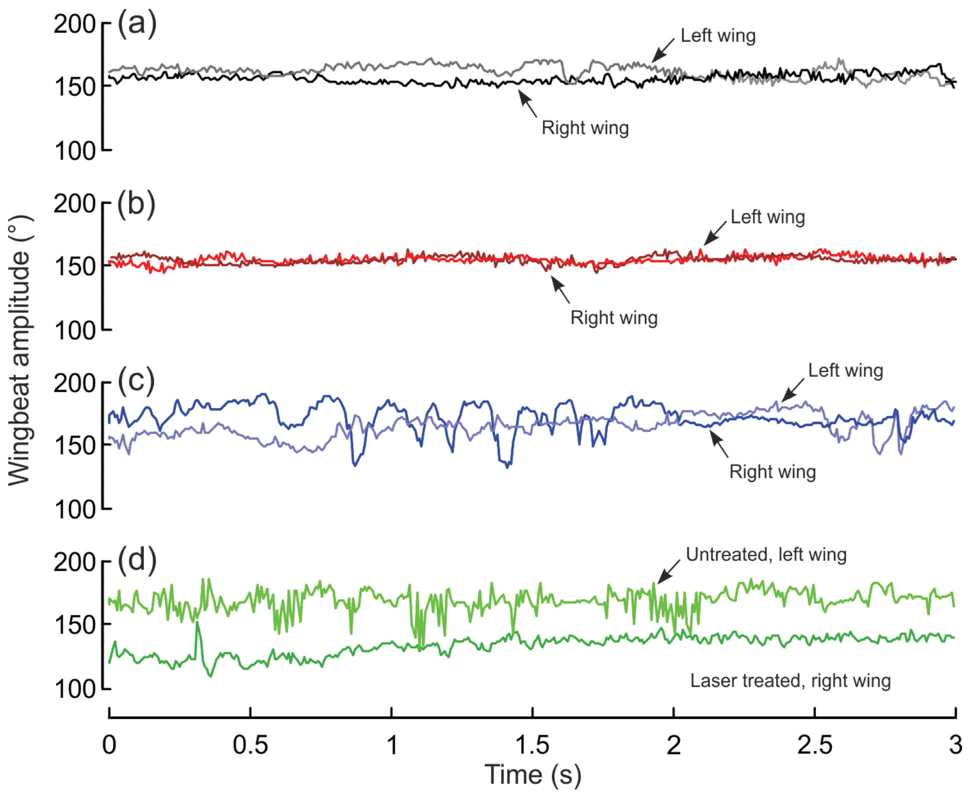

Supplementary Figure S1. Raw data traces of left and right wingbeat amplitude. (a) control fly, (b) haltere-immobilized fly, (c) wing nerve treated fly (35 mW), and (d) wing nerve damaged fly (90 mW).

b) Data sets

In the following, we provide the data underlying the summarizing figures in this study. More data will be provided upon request.

SD: standard deviation.

Figure 1f

Spikes counted per wing (N = 6).

Prior laser treatment spike count = 13, 39, 38, 27, 22, 14;

Post laser treatment spike count = 0, 5, 6, 8, 0, 0;

Figure 1g

Boxplot values, Means and SD. Wingbeat amplitude in degree.

90mW flies, non-treated wing: Median = 169.9, Maximum = 185, Minimum = 141.5, 75% Percentile = 173.9, 25% Percentile = 151.2, Mean = 163.3, SD = 14.7

90mW flies, treated wing: Median = 140.1, Maximum = 163.5, Minimum = 121.3, 75% Percentile = 149.1, 25% Percentile = 127.9, Mean = 141, SD = 14.3

Control flies: Median = 163.9, Maximum = 176.7, Minimum = 152.7, 75% Percentile = 167.2, 25% Percentile = 160.8, Mean = 164.5, SD = 8.8

35mW treated flies: Median = 169.8, Maximum = 182.9, Minimum = 145.4, 75% Percentile = 177.8, 25% Percentile = 145.35, Mean = 167, SD = 12.7

Haltere immobilized flies: Median = 160.12, Maximum = 185, Minimum = 153.4, 75% Percentile = 167.6, 25% Percentile = 151.2, Mean = 162, SD = 9.6

Figure 3c

Boxplot values, Means and SD. Full width half maximum of stripe distribution. Values in radiant (0 to 2pi).

Control flies:

Damping = 52

Median = 6.01, 25th Percentile = 1.093, Lower Adjacent = 0.819, Minimum = 0.819, 75th Percentile = 6.01, Upper Adjacent = 6.147, Maximum = 6.147, Number of outliers = 0, Mean = 4.0826, SD = 2.4905

Damping = 130

Median = 2.868, 25th Percentile = 2.08, Lower Adjacent = 0.819, Minimum = 0.819, 75th Percentile = 5.703, Upper Adjacent = 6.131, Maximum = 6.131, Number of outliers = 0, Mean = 3.6167, SD = 1.9478

Damping = 260

Median = 1.23, 25th Percentile = 1.093, Lower Adjacent = 0.683, Minimum = 0.683, 75th Percentile = 1.741, Upper Adjacent = 2.459, Maximum = 5.737, Number of outliers = 2 (Outlier values 5.736, 5.737), Mean = 1.6138, SD = 1.278

Damping = 520

Median = 0.956, 25th Percentile = 0.888, Lower Adjacent = 0.682, Minimum = 0.682, 75th Percentile = 1.093, Upper Adjacent = 1.366, Maximum = 2.186, Number of outliers = 2 (Outlier values 1.776, 2.186), Mean = 1.0602, SD = 0.3533

Damping = 1024

Median = 1.024, 25th Percentile = 1.093, Lower Adjacent = 0.683, Minimum = 0.683, 75th Percentile = 1.434, Upper Adjacent = 2.186, Maximum = 6.01, Number of outliers = 3 (Outlier values 4.508, 5.999, 6.01), Mean = 1.6334, SD = 1.5503

Haltere immobilized flies:

Damping = 52

Median = 4.439, 25th Percentile = 1.093, Lower Adjacent = 0.819, Minimum = 0.819, 75th Percentile = 6.01, Upper Adjacent = 6.147, Maximum = 6.147, Number of outliers = 0, Mean = 3.7758, SD = 2.481

Damping = 130

Median = 1.571, 25th Percentile = 1.161, Lower Adjacent = 0.956, Minimum = 0.956, 75th Percentile = 2.254, Upper Adjacent = 3.688, Maximum = 4.51, Number of outliers = 1 (Outlier value 4.51) , Mean = 1.8303, SD = 0.93

Damping = 260

Median = 1.093, 25th Percentile = 0.99, Lower Adjacent = 0.683, Minimum = 0.683, 75th Percentile = 1.5025, Upper Adjacent = 1.639, Maximum = 1.639, Number of outliers = 0, Mean = 1.164, SD = 0.2728

Damping = 520

Median = 1.366, 25th Percentile = 1.093, Lower Adjacent = 0.82, Minimum = 0.82, 75th Percentile = 1.776, Upper Adjacent = 1.912, Maximum = 1.912, Number of outliers = 0, Mean = 1.4001, SD = 0.3783

Damping = 1024

Median = 1.776, 25th Percentile = 1.639, Lower Adjacent = 1.229, Minimum = 1.229, 75th Percentile = 1.946, Upper Adjacent = 2.186, Maximum = 4.098, Number of outliers = 1 (Outlier value 4.098), Mean = 1.9123, SD = 0.6184

Wing nerve treated flies:

Damping = 52

Median = 3.961, 25th Percentile = 2.049, Lower Adjacent = 1.229, Minimum = 1.229, 75th Percentile = 5.873, Upper Adjacent = 6.01, Maximum = 6.01, Number of outliers = 0, Mean = 1.5366, SD = 1.9276

Damping = 130

Median = 5.464, 25th Percentile = 2.664, Lower Adjacent = 1.776, Minimum = 1.776, 75th Percentile = 6.01, Upper Adjacent = 6.28, Maximum = 6.28, Number of outliers = 0, Mean = 1.6903, SD = 1.8682

Damping = 260

Median = 2.459, 25th Percentile = 1.5025, Lower Adjacent = 0.683, Minimum = 0.683, 75th Percentile = 5.942, Upper Adjacent = 6.147, Maximum = 6.147, Number of outliers = 0, Mean = 3.5017, SD = 2.2736

Damping = 520

Median = 1.3

25th Percentile = 1.161, Lower Adjacent = 0.682, Minimum = 0.682, 75th Percentile = 2.186, Upper Adjacent = 3.55, Maximum = 3.55, Number of outliers = 0, Mean = 1.6903, SD = 0.9848

Damping = 1024

Median = 1.093, 25th Percentile = 0.812, Lower Adjacent = 0.682, Minimum = 0.682, 75th Percentile = 1.366, Upper Adjacent = 1.366, Maximum = 5.054, Number of outliers = 1 (Outlier value 5.054), Mean = 1.5366, SD = 1.4451

Figure 3d

Boxplot values, Means and SD. Absolute difference in wingbeat amplitude (values in degrees).

Control flies

Damping = 52

Median = 14.95, 25th Percentile = 10.9, Lower Adjacent = 5.87, Minimum = 5.87, 75th Percentile = 27.68, Upper Adjacent = 35.37, Maximum = 83.97, Number of outliers = 3 (Outlier values 60.0, 73.91, 83.97) , Mean = 24.9, SD = 23.42

Damping = 130

Median = 17.23, 25th Percentile = 10.49, Lower Adjacent = 10.49, Minimum = 5.88, 75th Percentile = 24.04, Upper Adjacent = 33.79, Maximum = 57.41, Number of outliers = 1 (Outlier value 57.41) , Mean = 18.85, SD = 11.68

Damping = 260

Median = 11.48, 25th Percentile = 8.76, Lower Adjacent = 5.54, Minimum = 5.54, 75th Percentile = 19.78, Upper Adjacent = 31.72, Maximum = 81.54, Number of outliers = 1 (Outlier value 81.54) , Mean = 16.34, SD = 14.74

Damping = 520

Median = 11.27, 25th Percentile = 8.2, Lower Adjacent = 6.77, Minimum = 6.77, 75th Percentile = 15.47, Upper Adjacent = 20.16, Maximum = 20.16, Number of outliers = 0, Mean = 11.9, SD = 3.84

Damping = 1024

Median = 13.36, 25th Percentile = 10.5, Lower Adjacent = 7.6, Minimum = 7.6, 75th Percentile = 14.91, Upper Adjacent = 20.15, Maximum = 20.15, Number of outliers = 0, Mean = 12.96, SD = 2.92

Haltere immobilized flies:

Damping = 52

Median = 10.55, 25th Percentile = 7.922, Lower Adjacent = 4.8, Minimum = 4.8, 75th Percentile = 16.87, Upper Adjacent = 21.53, Maximum = 21.53, Number of outliers = 0, Mean = 12.11, SD = 5.33

Damping = 130

Median = 9.05, 25th Percentile = 7.74, Lower Adjacent = 4.45, Minimum = 4.45, 75th Percentile = 11.94, Upper Adjacent = 16.65, Maximum = 19.37, Number of outliers = 1 (Outlier value 19.37), Mean = 10.03, SD = 3.78

Damping = 260

Median = 8.49, 25th Percentile = 7.33, Lower Adjacent = 4.5, Minimum = 4.5, 75th Percentile = 11.62, Upper Adjacent = 18.03, Maximum = 18.03, Number of outliers = 0, Mean = 9.22, SD = 3.58

Damping = 520

Median = 11.27, 25th Percentile = 8.2, Lower Adjacent = 6.77, Minimum = 6.77, 75th Percentile = 15.47, Upper Adjacent = 20.16, Maximum = 20.16, Number of outliers = 0, Mean = 10.24, SD = 3.23

Damping = 1024

Median = 10.09, 25th Percentile = 7.82, Lower Adjacent = 5.44, Minimum = 5.44, 75th Percentile = 12.29, Upper Adjacent = 16.36, Maximum = 16.36, Number of outliers = 0, Mean = 9.98, SD = 3.04

Wing nerve treated flies:

Damping = 52

Median = 31.29, 25th Percentile = 23.41, Lower Adjacent = 10.94, Minimum = 10.94, 75th Percentile = 36.67, Upper Adjacent = 53.87, Maximum = 60.2, Number of outliers = 1 (Outlier value 60.2), Mean = 32.42, SD = 15.75

Damping = 130

Median = 29.61, 25th Percentile = 19.48, Lower Adjacent = 13.35, Minimum = 13.35, 75th Percentile = 50.38, Upper Adjacent = 78.19, Maximum = 78.19, Number of outliers = 0, Mean = 36.32, SD = 22.55

Damping = 260

Median = 24.2, 25th Percentile = 8.76, Lower Adjacent = 10.21, Minimum = 10.21, 75th Percentile = 35.04, Upper Adjacent = 57.23, Maximum = 110.72, Number of outliers = 1 (Outlier value 110.72), Mean = 33.51, SD = 28.86

Damping = 520

Median = 17.38, 25th Percentile = 15.49, Lower Adjacent =10.69, Minimum = 10.69, 75th Percentile = 20.92, Upper Adjacent = 20.92, Maximum = 45.41, Number of outliers = 1 (Outlier value 45.41), Mean = 20.46, SD = 10.63

Damping = 1024

Median = 25.86, 25th Percentile = 17.76, Lower Adjacent = 11.88, Minimum = 11.88, 75th Percentile = 36.47, Upper Adjacent = 41.54, Maximum = 41.54, Number of outliers = 0, Mean = 26.70, SD = 11.11

Figure 4b

Boxplot values, Means and SD. Sum of wingbeat amplitudes in degrees.

Maxima:

Control flies

Median = 346.3, 25th Percentile = 337.8, Lower Adjacent = 334.2, Minimum = 307.1, 75th Percentile = 349.1, Upper Adjacent = 360, Maximum = 360, Number of outliers = 2 (Outlier values 307.1, 319.5), Mean = 344, SD = 12.5

Haltere immobilized flies

Median = 339.7, 25th Percentile = 321.8, Lower Adjacent = 312.2, Minimum = 312.2, 75th Percentile = 347.9, Upper Adjacent = 360, Maximum = 360, Number of outliers = 0, Mean = 336.7, SD = 15.79

Wing nerve ablated flies

Median = 358.7, 25th Percentile = 339.3, Lower Adjacent = 333.3, Minimum = 333.3, 75th Percentile = 360, Upper Adjacent = 360, Maximum = 360, Number of outliers = 0, Mean = 350.4, SD = 11.4

Minima:

Control flies

Median = 306.7, 25th Percentile = 287.7, Lower Adjacent = 272.3, Minimum = 272.3, 75th Percentile = 311.7, Upper Adjacent = 340.4, Maximum = 340.4, Number of outliers = 0, Mean = 301.6, SD = 15.8

Haltere immobilized flies

Median = 303.2, 25th Percentile = 294.3, Lower Adjacent = 271.4, Minimum = 271.4, 75th Percentile = 314, Upper Adjacent = 341.3, Maximum = 358.7, Number of outliers = 1 (Outlier value 358.7) , Mean = 305.6, SD = 20

Wing nerve ablated flies

Median = 276.4, 25th Percentile = 273.1, Lower Adjacent = 270.7, Minimum = 270.7, 75th Percentile = 297.6, Upper Adjacent = 307.6, Maximum = 307.6, Number of outliers = 0, Mean = 283.6, SD = 15.2

Figure 4c

Boxplot values, Means and SD. Wingbeat frequency in Hertz.

Maxima:

Control flies

Median = 218.1, 25th Percentile = 207.1, Lower Adjacent = 180.4, Minimum = 180.4, 75th Percentile = 226, Upper Adjacent = 237.6, Maximum = 261.3, Number of outliers = 1 (Outlier value 261.3) , Mean = 217.6, SD = 16.1

Haltere immobilized flies

Median = 218.1, 25th Percentile = 213.7, Lower Adjacent = 197.6, Minimum = 197.6, 75th Percentile = 226.5, Upper Adjacent = 243.1, Maximum = 243.1, Number of outliers = 0, Mean = 219.4, SD = 11.3

Wing nerve ablated flies

Median = 213.7, 25th Percentile = 212, Lower Adjacent = 208.8, Minimum = 208.8, 75th Percentile = 222.7, Upper Adjacent = 231.9, Maximum = 263.2, Number of outliers = 1 (Outlier value 263.2) , Mean = 221.2, SD = 17.2

Minima:

Control flies

Median = 196.1, 25th Percentile = 189.1, Lower Adjacent = 173, Minimum = 150, 75th Percentile = 203.6, Upper Adjacent = 215.2, Maximum = 215.2, Number of outliers = 1 (Outlier value 150) , Mean = 194.3, SD = 14.3

Haltere immobilized flies

Median = 198.3, 25th Percentile = 192.2, Lower Adjacent = 184.1, Minimum = 166.2, 75th Percentile = 203.9, Upper Adjacent = 220.1, Maximum = 220.1, Number of outliers = 1 (Outlier value 166.2) , Mean = 198.5, SD = 11.5

Wing nerve ablated flies

Median = 183.3, 25th Percentile = 174.1, Lower Adjacent = 165.2, Minimum = 165.2, 75th Percentile = 191.8, Upper Adjacent = 195.6, Maximum = 195.6, Number of outliers = 0, Mean = 182.7, SD = 10.2

Figure 4d

Boxplot values, Means and SD. Values in degrees / Hertz.

Wingbeat amplitude range:

Control flies

Median = 17.5, 25th Percentile = 15.5, Lower Adjacent = 9.8, Minimum = 9.8, 75th Percentile = 25.7, Upper Adjacent = 38.3, Maximum = 38.3, Number of outliers = 0, Mean = 42.4, SD = 15.5

Haltere immobilized flies

Median = 15.2, 25th Percentile = 9.35, Lower Adjacent = 1.3, Minimum = 1.3, 75th Percentile = 17.1, Upper Adjacent = 26.8, Maximum = 34.1, Number of outliers = 1 (Outlier value 34.1) , Mean = 31.1, SD = 15.1

Wing nerve ablated flies

Median = 34.2, 25th Percentile = 25.4, Lower Adjacent = 17.7, Minimum = 17.7, 75th Percentile = 42.1, Upper Adjacent = 43.4, Maximum = 43.4, Number of outliers = 0, Mean = 66.8, SD = 19.4

Wingbeat frequency range:

Control flies

Median = 18.1, 25th Percentile = 17, Lower Adjacent = 14.2, Minimum = 14.2, 75th Percentile = 26, Upper Adjacent = 30.4, Maximum = 71.6, Number of outliers = 2 (Outlier values 47.9, 71.6) , Mean = 23.3, SD = 12.4

Haltere immobilized flies

Median = 20.1, 25th Percentile = 16.2, Lower Adjacent = 10.8, Minimum = 1.3, 75th Percentile = 17.1, Upper Adjacent = 31.4, Maximum = 38.9, Number of outliers = 1 (Outlier value 38.9) , Mean = 20.9, SD = 7

Wing nerve ablated flies

Median = 27.5, 25th Percentile = 23.4, Lower Adjacent = 17.7, Minimum = 17.7, 75th Percentile = 46.8, Upper Adjacent = 50.6, Maximum = 89.1, Number of outliers = 1 (Outlier value 89.1) , Mean = 38.5, SD = 22.5
